# Gesundheitliche Lage älterer Menschen in stationärer Langzeitpflege – ein narratives Review

**DOI:** 10.1007/s00103-026-04283-x

**Published:** 2026-08-07

**Authors:** Christina Hoffmann, Gabriele Meyer, Jens Abraham

**Affiliations:** Institut für Gesundheits‑, Hebammen- und Pflegewissenschaft, Medizinische Fakultät der Martin-Luther-Universität Halle-Wittenberg, 06112 Halle (Saale), Deutschland

**Keywords:** Gesundheitliche Lage, Interventionen, Narratives Review, Pflegeheim, Qualitätsberichterstattung, Health situation, Interventions, Narrative review, Nursing home, Quality reporting

## Abstract

Mit zunehmendem Alter steigt das Risiko für gesundheitliche Beeinträchtigungen und Pflegebedürftigkeit. Etwa 14 % der pflegebedürftigen Menschen in Deutschland werden in Pflegeheimen versorgt; unter den Pflegeheimbewohner:innen beträgt der Anteil hochaltriger Menschen etwa 70 %. Pflegeheimbewohner:innen sind durch vielfältige Problemstellungen besonders vulnerabel. Die Datenlage zum Gesundheitszustand dieser Personengruppe ist jedoch unzureichend. Dieses narrative Review fasst die empirischen Befunde aus Deutschland der letzten 10 Jahre zur gesundheitlichen Lage hochaltriger Menschen in Pflegeheimen zusammen. Zudem stellt es Interventionsansätze und aktuelle Berichte zur Versorgungsqualität dar.

Im November 2025 wurde eine systematische Recherche in MEDLINE via PubMed durchgeführt, ergänzt durch eine freie Suche nach Qualitätsberichten. Die Recherche ergab insgesamt 1597 Treffer, 76 Publikationen wurden final eingeschlossen. Die Studien waren hinsichtlich der untersuchten Themenfelder und in ihrer Berichterstattung sehr heterogen. Thematische Schwerpunkte waren Hautgesundheit, Medikation sowie herausfordernde Verhaltensweisen bei Menschen mit Demenz, wobei sich teilweise erhebliche Unterschiede hinsichtlich berichteter Häufigkeiten zeigten. Relevante Aspekte wie Schmerz, Ernährung, Mundgesundheit und Lebensqualität wurden nicht oder kaum adressiert. Es lagen nur wenige Berichte vor, die sich mit der Versorgungsqualität befassen.

Insgesamt ist die gesundheitliche Lebenssituation hochaltriger Menschen in Pflegeeinrichtungen bislang unzureichend beschrieben. Es besteht Bedarf an methodisch robusten Studien mit angemessenen Erhebungsmethoden und Ergebnismaßen, um die spezifischen Bedarfslagen dieser Zielgruppe umfassend zu erfassen und zu berichten.

**Zusatzmaterial online:**

Zusätzliche Informationen sind in der Online-Version dieses Artikels (https://doi.org/10.1007/s00103-026-04283-x) enthalten.

## Abstract (en)

The risk of health impairments and the need for care increases with age. Approximately 14 % of people in need of care in Germany are cared for in nursing homes; among nursing home residents, the proportion of very old people is around 70 %. Nursing home residents are particularly vulnerable due to a variety of issues, but the data available on the health status of this target group are insufficient. This narrative review summarizes the empirical findings from Germany over the past decade on the health status of very old people in nursing homes. Furthermore, it presents intervention approaches and current reports on the quality of care.

A systematic search was conducted in MEDLINE via PubMed in November 2025 and supplemented by a free search for quality reports. The search yielded a total of 1597 hits, and 76 publications were ultimately included. The studies were very heterogeneous in terms of topics and reporting. The main topics were skin health, medication, and challenging behavior in people with dementia, with some marked differences in reported frequencies. Relevant aspects such as pain, nutrition, oral health, and quality of life were not addressed or only rarely. Only a few reports were available on the quality of care.

Overall, the health situation of very old people in nursing homes has not been adequately described to date. There is a need for methodologically robust studies with appropriate data collection methods and outcome measures in order to comprehensively assess and report on the specific needs of this target group.

## Einleitung

Mit zunehmendem Alter steigt das Risiko für gesundheitliche Beeinträchtigungen, insbesondere für chronische Erkrankungen, Multimorbidität und Funktionseinbußen. Zu den häufigsten Erkrankungen hochaltriger Menschen zählen Herz-Kreislauf-Erkrankungen, Diabetes mellitus, Krebserkrankungen, chronische Lungenerkrankungen und Erkrankungen des Muskel-Skelett-Systems [1]. Zu den häufigsten psychiatrischen Diagnosen im höheren Lebensalter zählt die Demenz. Hochaltrige Menschen sind zudem häufiger von Gebrechlichkeit (*Frailty*) und Multimorbidität betroffen [2].

Ebenso steigt mit zunehmendem Alter das Risiko, pflegebedürftig zu sein. So waren im Jahr 2023 in der Altersgruppe der 75- bis 79-Jährigen etwa 18 % pflegebedürftig. In der Altersgruppe der 80- bis 84-Jährigen lag der Anteil bereits bei etwa 35 %. In der Altersgruppe ab 90 Jahren war der Anteil mit 87 % am höchsten [3]. Etwa 14 % der knapp 5,7 Mio. pflegebedürftigen Personen werden in den ca. 16.500 Einrichtungen [4] der stationären Langzeitpflege in Deutschland versorgt [5]. Der Anteil der hochaltrigen Menschen ab 80 Jahren beträgt ca. 70 % [6]. Pflegeheimbewohner:innen sind aufgrund vielfältiger Problemlagen besonders vulnerabel. Dazu zählen Polypharmazie, Mobilitätseinschränkungen, Sturzgefährdung, Flüssigkeitsmangel und Mangelernährung, Einschränkungen der Mundgesundheit, Auftreten chronischer Wunden, kognitive Einschränkungen, herausfordernde Verhaltensweisen bei Demenz und Risiko für Hospitalisierung. Die sich daraus ergebenden Versorgungsbedarfe sind hochkomplex.

Der Neunte Altersbericht der Bundesregierung betont, dass die verfügbare Datenbasis zum Gesundheitszustand von hochaltrigen Menschen unbefriedigend ist und insbesondere über die Bewohner:innen von Langzeitpflegeeinrichtungen wenig bekannt ist [7, 8]. Methodische Herausforderungen werden angeführt. Der Zugang zu Pflegeheimen sowie die Rekrutierung und der Einbezug der kognitiv und physisch oftmals stark beeinträchtigten Bewohner:innen sind herausfordernd [7–9].

Ziel unseres narrativen Reviews ist es, die empirischen Befunde der letzten 10 Jahre zur gesundheitlichen Lage hochaltriger Menschen in Pflegeheimen in Deutschland zusammenzustellen sowie einen Überblick über publizierte Interventionen zur Verbesserung der gesundheitlichen Lage zu geben. Aktuelle Berichte, die sich mit der Versorgungsqualität im Pflegeheim befassen, werden exemplarisch vorgestellt.

## Methodisches Vorgehen

Die systematische Literaturrecherche erfolgte im November 2025 in der Datenbank MEDLINE via PubMed. Die Suche war auf den Zeitraum der letzten 10 Jahre sowie auf englisch- und deutschsprachige Publikationen begrenzt. Es wurde eine gemeinsame Recherche nach Studien zur Beschreibung der gesundheitlichen Lage der Pflegeheimbewohner:innen und zu den Interventionsstudien durchgeführt. Hierfür wurde ein sensitiver Rechercheansatz gewählt. Es wurde ein Suchkomplex zum Setting „Pflegeheim“ verwendet, wobei folgende Suchbegriffe unter Nutzung von Trunkierungen kombiniert wurden: „nursing home“, „long term care facility“, „home for the aged“, „care home“, „residential care“, „residential care facility“, „residential facilities“. Da der Fokus auf Studien aus Deutschland lag, wurde dieser Suchkomplex mit einem validierten geografischen Filter zur Identifikation von Studien aus Deutschland kombiniert [10]. Thematisch relevante Reviews wurden zusätzlich nach potenziell geeigneten Primärstudien durchsucht. Eine Übersicht der Ein- und Ausschlusskriterien findet sich in der Tab. 1.

**Tab. 1:** Ein- und Ausschlusskriterien der Literaturrecherche

Domäne	Einschlusskriterien	Ausschlusskriterien
**Designs**	*Deskription der gesundheitlichen Lage*	Letter to the editor, Expert:innenmeinungen, Editorials, Studienprotokolle, qualitative Studien, Prozessevaluation, Methodenreport, Entwicklung/Testung von Assessmentinstrumenten, systematische Literaturübersichten (Rapid Reviews, Scoping-Reviews, Systematic Reviews)
	Beobachtungsstudien, deskriptive Studien, Analysen von Routinedaten/Sekundärdatenanalysen	
	*Interventionsstudien*	
	(Cluster-)randomisierte kontrollierte Studien	
**Population**	Hochaltrige Menschen im Pflegeheim	Kinder und Jugendliche, erwachsene Personen, Studien, die sich ausschließlich auf Personal der Pflegeheime beziehen
**Land**	Deutschland, multinationale Studien mit separater Berichterstattung für Deutschland	Alle anderen Länder
**Sprache**	Deutsch, Englisch	Andere Sprachen
**Setting(s)**	Stationäre Langzeitpflegeeinrichtungen	Akutstationäre Versorgung, Rehabilitation, Tagespflege, ambulante Pflege, teilstationäre Pflege, Hospiz, Einrichtungen für Menschen mit Behinderung, Betreutes Wohnen, Wohngemeinschaften, Psychiatrie
**Publikationsjahre**	Letzten 10 Jahre (Recherche erfolgte im November 2025)	Älter als 10 Jahre

Das Titel- und Abstract-Screening wurde zwischen zwei Personen hälftig aufgeteilt (CH und JA). Für 10 % der Treffer wurde eine doppelte Kontrolle durchgeführt mit Konsensfindung bei abweichenden Einschätzungen. Infrage kommende Publikationen wurden anschließend im Volltext geprüft. Da das Ziel des narrativen Reviews in der zusammenfassenden Darstellung der vorhandenen Forschungsliteratur zur Thematik liegt und nicht in der Bewertung der Wirksamkeit einzelner Interventionen und deren Aussagekraft, wurde auf eine Bewertung der methodischen Güte der eingeschlossenen Studien verzichtet.

Aus den eingeschlossenen Studien wurde neben allgemeinen Merkmalen der thematische Fokus erfasst und anschließend nach verschiedenen übergeordneten Kategorien systematisiert. Für die Deskription der gesundheitlichen Lage der Bewohner:innen wurden zusätzlich die berichteten epidemiologischen Kennzahlen (u. a. Prävalenz, Inzidenz, Mortalitätsrate) entnommen. Die Synthese der Ergebnisse erfolgte narrativ.

Neben der Studienrecherche erfolgte eine freie Recherche nach aktuellen Berichten, die im Zeitraum der letzten fünf Jahre veröffentlicht wurden und sich mit der Versorgungsqualität in Pflegeheimen auf überregionaler Ebene befassen. Diese wurden hinsichtlich Hintergrunds, methodischer Aspekte und thematischer Schwerpunkte gesichtet und werden nur exemplarisch beschrieben.

## Ergebnisse

**Abb. 1:**
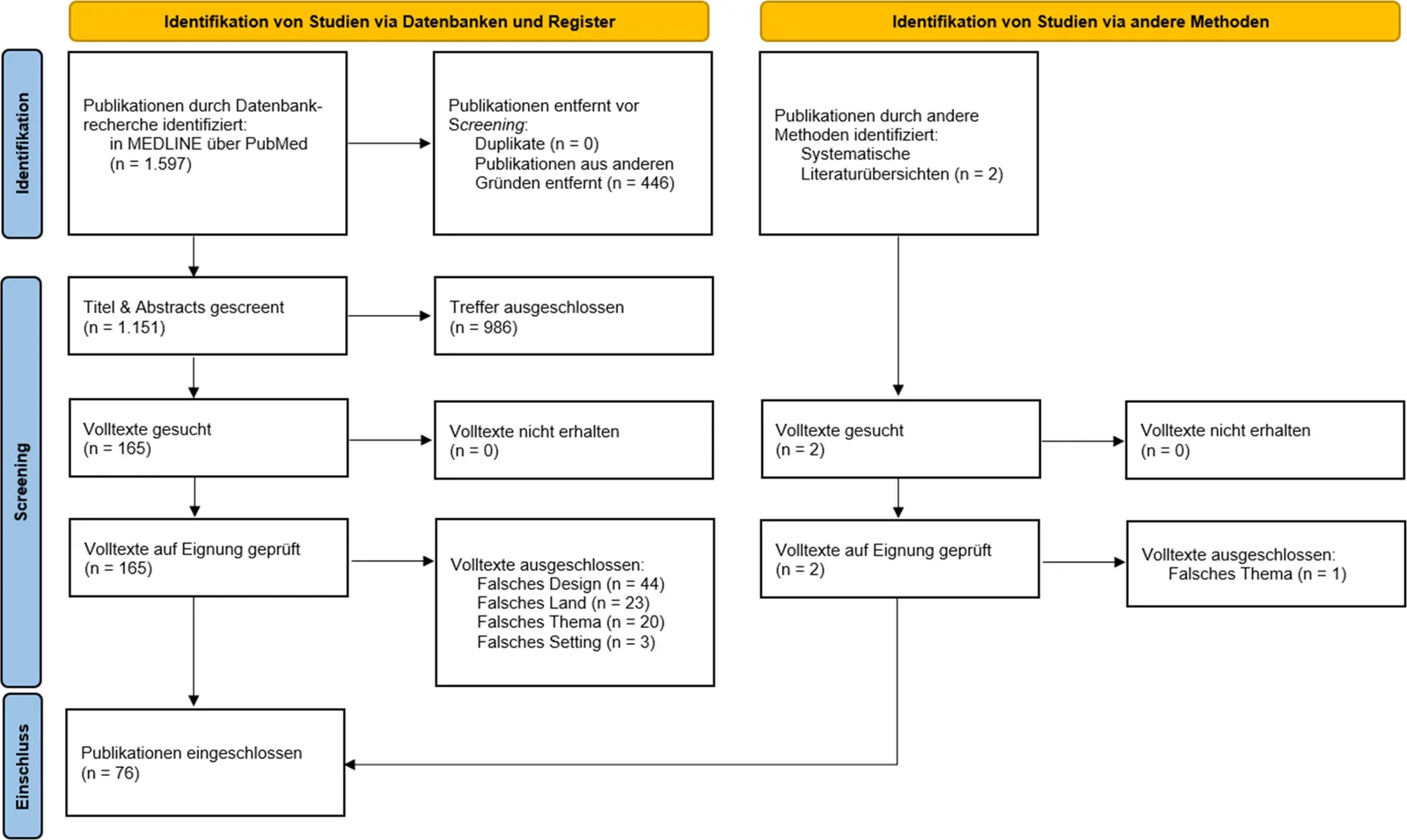
Verlauf des Rechercheprozesses

Die Datenbankrecherche ergab insgesamt 1597 Treffer. In einem ersten Schritt wurden nach Ausschluss irrelevanter Treffer 1151 Publikationen im Titel-Abstract-Screening gesichtet. Insgesamt 165 Studien wurden im Volltext bezüglich des Einschlusses geprüft. Alle systematischen Literaturübersichten wurden ebenfalls nach geeigneten Arbeiten durchgesehen. Hierbei gab es eine große Überschneidung mit der eigenen Recherche und ein Großteil der Primärstudien war aufgrund der Publikationszeiträume und falscher Länder nicht geeignet. Zur Beschreibung der gesundheitlichen Lage der Bewohner:innen wurden letztlich 53 Publikationen eingeschlossen, bei den Interventionsansätzen waren es insgesamt 23 Publikationen (Abb. 1).

Es zeigte sich eine große Heterogenität bei der Anzahl der untersuchten Einrichtungen und Bewohner:innen. Bei Studien zur Beschreibung der gesundheitlichen Lage der Bewohner:innen wurden zwischen 3 und 280 Einrichtungen einbezogen. Die Stichprobengröße variierte zwischen 63 und 259.328 Bewohner:innen. Die Teilnehmenden waren zwischen 76,5 und 90,2 Jahre alt und überwiegend weiblich. Bei Interventionsstudien variierte die Stichprobengröße zwischen einer Einrichtung und 120 Pflegeheimen sowie zwischen 46 und 8800 teilnehmenden Bewohner:innen, die zwischen 80 und 88 Jahren alt und ebenfalls mehrheitlich weiblich waren. Eine umfassende Übersicht der Studien findet sich im Onlinematerial 1 und 2 (eTab. 1 und 2).

## Gesundheitliche Lage der Bewohner:innen

**Abb. 2:**
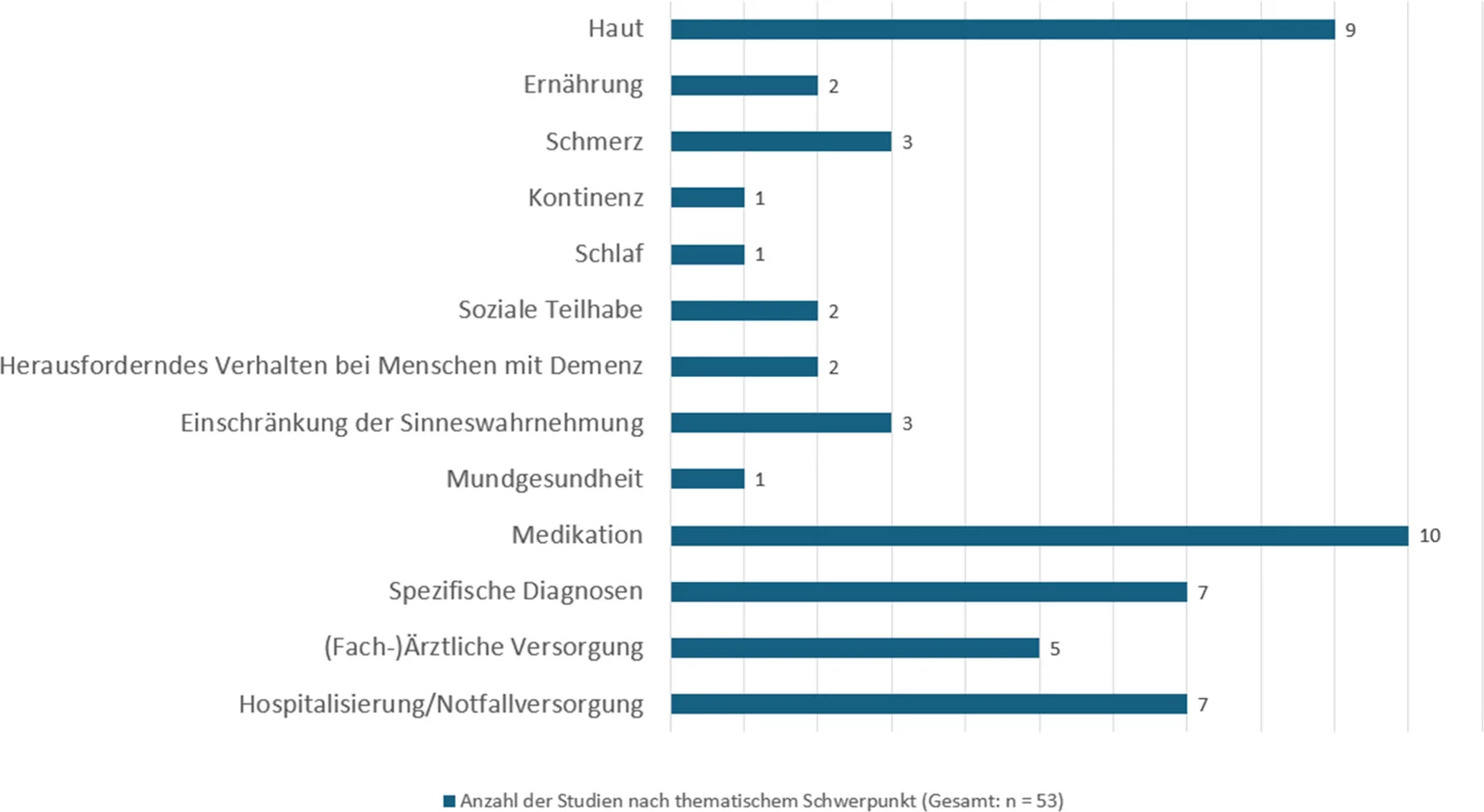
Thematische Schwerpunkte zur gesundheitlichen Lage der Bewohner:innen

Es zeigte sich eine große Bandbreite an untersuchten Themen- und Problemfeldern. Insgesamt 13 thematische Kategorien wurden identifiziert. Am häufigsten wurden Studien durchgeführt, die sich mit der Medikation befasst haben, mit dem Hautzustand, mit spezifischen Krankheitsdiagnosen und Syndromen sowie Hospitalisierung und Notfallversorgung. Abb. 2 zeigt einen Überblick über die thematischen Schwerpunkte der Studien.

### Hautgesundheit.

In 9 Studien wurden der Hautzustand bzw. die Häufigkeit verschiedener Hautveränderungen und Hauterkrankungen erhoben. Neben der Häufigkeit von Vorstufenläsionen (Vorstufen von Hautkrebs) und Hautkrebs [11] wurden sowohl das Vorliegen einer Inkontinenz-assoziierten Dermatitis (IAD; durch Inkontinenz ausgelöste Hautreizungen; [12]) als auch Intertrigo (wunde Hautstellen in Hautfalten; [13]) und chronische Wunden [14] untersucht. Die Prävalenzen von Intertrigo variierten dabei zwischen den Studien mit 16 % [13] und 35 % [15]. Auch beim Vorliegen von IAD wurden unterschiedliche Prävalenzen berichtet. Boronat-Garrido et al. [12] erfassten einen Anteil von 5 %, während bei Völzer et al. [15] 21 % der Bewohner:innen eine IAD aufwiesen. Chronische Wunden lagen bei knapp 8 % der Teilnehmenden vor [14]. Die Dekubitus-Prävalenz wurde in 2 Studien erhoben und lag jeweils unter 5 % [16, 17]. In 3 Studien lag der primäre Fokus auf der Erhebung von Hauttrockenheit [16, 18, 19]. Dabei konnte in 2 Studien bei etwa der Hälfte der Teilnehmenden Hauttrockenheit festgestellt werden [16, 18], in der dritten Studie waren nahezu alle Teilnehmenden betroffen ([19]; vgl. eTab. 1 im Onlinematerial 1).

### Ernährung.

In Bezug auf die Ernährung wurde in der Studie von Streicher et al. [20] die Einnahme von oralen Nahrungsergänzungsmitteln erhoben. Dabei zeigte sich, dass 8 % der Teilnehmenden orale Nahrungsergänzungsmittel erhalten haben. Strube-Lahrmann et al. [21] untersuchten die Häufigkeit von Untergewicht, wobei der Anteil bei 14 % lag.

### Schmerz.

Eine Studie untersuchte die Schmerzintensität bei Personen mit chronischen Schmerzen [22]. Ein Drittel der Teilnehmenden litt unter starken Schmerzen (31 %). Centmayer et al. [23] haben die Prävalenz von Schmerzen erhoben – etwa 32 % der Bewohner:innen gaben Schmerzen an. Eine weitere Studie befasste sich neben der Prävalenz von Rückenschmerzen mit dem Vorliegen von Schwindel, Schlafstörungen sowie der generellen Einschätzung der Gesundheit [24]. Dabei gaben 63 % an, unter Rückenschmerzen zu leiden und 45 % schätzten ihre allgemeine Gesundheit als schlecht ein.

### Schlaf.

Lediglich eine Studie [25] beschäftigte sich mit dem Thema Schlaf bei Bewohner:innen mit Demenz. In der multizentrischen Querschnittsstudie lag der Anteil der Bewohner:innen mit Schlafstörungen bei 23 %.

### Kontinenz.

Zum Thema Kontinenz bzw. Ausscheidung gab es ebenfalls nur eine Studie [26]. Dabei wurde die Häufigkeit des Einsatzes von Blasendauerkathetern erhoben. Von insgesamt 852 Bewohner:innen hatten etwa 13 % einen Blasendauerkatheter.

### Soziale Teilhabe.

Zwei Arbeiten fokussierten sich auf das Thema soziale Teilhabe. Eine Untersuchung analysierte die Häufigkeiten sozialer Interaktionen von Menschen mit Demenz sowie die Interaktionspartner:innen [27]. Bei rund 72 % konnten im Beobachtungszeitraum keine sozialen Interaktionen festgestellt werden. Interaktionen fanden primär mit Mitarbeitenden der Einrichtung und anderen Bewohner:innen statt. Thematischer Fokus bei Hajek et al. [28] war die Erfassung von Einsamkeit. Hier wurde bei 26 % der Teilnehmenden eine moderate und bei etwa 18 % stark ausgeprägte Einsamkeit ermittelt.

### Herausforderndes Verhalten bei Menschen mit Demenz.

Das Auftreten von herausfordernden Verhaltensweisen bei Menschen mit Demenz wurde in 2 Studien untersucht. Als Ergebnis haben Hüsken et al. [29] die Prävalenz von verschiedenen sogenannten neuropsychiatrischen Symptomen, wie Erregungszustände (36 %), Depression (33 %) und Apathie (33 %), erhoben. Bei Palm et al. [30] wurde die Prävalenz von agitiertem Verhalten systematisch erfasst und lag bei rund 6 %.

### Einschränkung der Sinneswahrnehmung.

Die Häufigkeiten von Augenerkrankungen und Sehbeeinträchtigungen wurden in 3 Studien untersucht [31–33]. Bei rund 17 % der Bewohner:innen wurde eine moderate oder schwere Sehbeeinträchtigung festgestellt [32]. Thederan et al. [31] stellten bei 22 % der Teilnehmenden einen akuten augenärztlichen Behandlungsbedarf fest. In der Studie von Bock et al. [33] wurden die Hörsituation und Hörversorgung im Pflegeheim untersucht. Es wurde berichtet, dass trotz einer Hörgeräteindikation 29 % der Teilnehmenden kein Hörgerät besaßen.

### Mundgesundheit.

Untersuchungsgegenstand einer Studie war die mundgesundheitsbezogene Lebensqualität der Bewohner:innen [34]. Bei 41 % der Bewohner:innen wurde dabei eine Beeinträchtigung festgestellt.

### Medikation.

Bezüglich der Medikation wurden unterschiedliche Aspekte wie Polypharmazie [35] und die Verordnungshäufigkeit von Psychopharmaka [36, 37] untersucht. Bei 35 % der Teilnehmenden mit und ohne Demenz waren Antipsychotika verordnet [36]. Eine auf Versicherungsdaten basierende Studie fokussierte sich auf die Verordnungshäufigkeit von Psychopharmaka mit sedierender und potenziell freiheitseinschränkender Wirkung [38]. Es zeigte sich, dass 55 % der Bewohner:innen mindestens ein Psychopharmakon als Regelmedikation erhielten und 34 % ein sedierendes Regelmedikament.

Der Einsatz von potenziell inadäquater Medikation (PIM; [39, 40]) sowie von Medikamenten mit fraglichem Nutzen bei Personen mit und ohne Demenz [41] wurde ebenfalls untersucht. Dabei wurden sehr unterschiedliche Häufigkeiten berichtet. In einer Studie lag der Anteil der Bewohner:innen mit mindestens einer verordneten PIM bei 26 % [39]. Bei Makan et al. [40] lag der Anteil bei 84 %. Zudem wurde der Einsatz von Schmerzmedikamenten untersucht, wobei bei 74 % der Teilnehmenden eine Verordnung von mindestens einem Analgetikum vorlag [42]. Andere Studien betrachteten die Einnahme von Medikamenten zur Prävention von kardiovaskulären Ereignissen [43] sowie die pharmakologische Osteoporoseprävention ([44]; vgl. eTab. 1 im Onlinematerial 1).

### Spezifische Diagnosen.

Sieben Studien untersuchten das Auftreten verschiedener Krankheitsbilder und Syndrome. Neben der Inzidenz von Pneumonie [45] wurde die Prävalenz von Niereninsuffizienz [46] und Parkinson [47] bei den Bewohner:innen erfasst. Eine weitere Arbeit befasste sich mit dem Auftreten von Hüftfrakturen spezifisch bei Bewohner:innen mit und ohne multiple Sklerose [48]. Darüber hinaus wurden die Häufigkeiten von *Frailty* [49], sitzendem Verhalten und Sarkopenie [50] sowie Dysphagie [51] untersucht. Die berichteten Ergebnismaße in den Studien waren sehr heterogen (vgl. eTab. 1 im Onlinematerial 1).

### (Fach‑)Ärztliche Versorgung.

Neben der Inanspruchnahme zahnmedizinischer Leistungen [52] wurde die Nutzung hausärztlicher und weiterer fachärztlicher Versorgung [53, 54] untersucht. In allen 3 Studien wurden hausärztliche, zahnärztliche und neurologische Leistungen am häufigsten in Anspruch genommen. Eine Studie analysierte augenärztliche Bedarfe und Versorgung [55]. Bei 61 % der Bewohner:innen wurden behandlungsbedürftige Befunde festgestellt. In einer weiteren Studie wurde die fachärztliche Unterversorgung bzgl. Seh- und Hörfähigkeit, Mundgesundheit und des Parkinson-Syndroms erhoben, wobei vor allem fachärztlicher Versorgungsbedarf im Bereich Sehfähigkeit bestand [56].

### Hospitalisierung und Notfallversorgung.

Häufig wurden die Inanspruchnahme akuter medizinischer Leistungen, darunter ärztliche Versorgung außerhalb regulärer Sprechzeiten, Notaufnahmen (ohne stationäre Aufnahme), akute Krankenhauseinweisungen sowie Krankenhausaufenthalte und Kontakte zu regionalen ärztlichen Bereitschaftsdiensten untersucht [57–63]. Die Prävalenz von Krankenhauseinweisungen lag bei 46 % [63]. Zwei Studien fokussierten dabei spezifisch auf Hospitalisierungen in der letzten Lebensphase ([60, 61]; eTab. 1 im Onlinematerial 1). In weiteren Arbeiten wurde die Hospitalisierung spezifisch nach Geschlecht untersucht [59] sowie für neu aufgenommene Bewohner:innen mit und ohne Demenz [57]. Die Häufigkeit von potenziell vermeidbaren Krankenhausaufenthalten lag bei knapp einem Drittel [62].

## Interventionsansätze zur Verbesserung der gesundheitlichen Lage

**Abb. 3:**
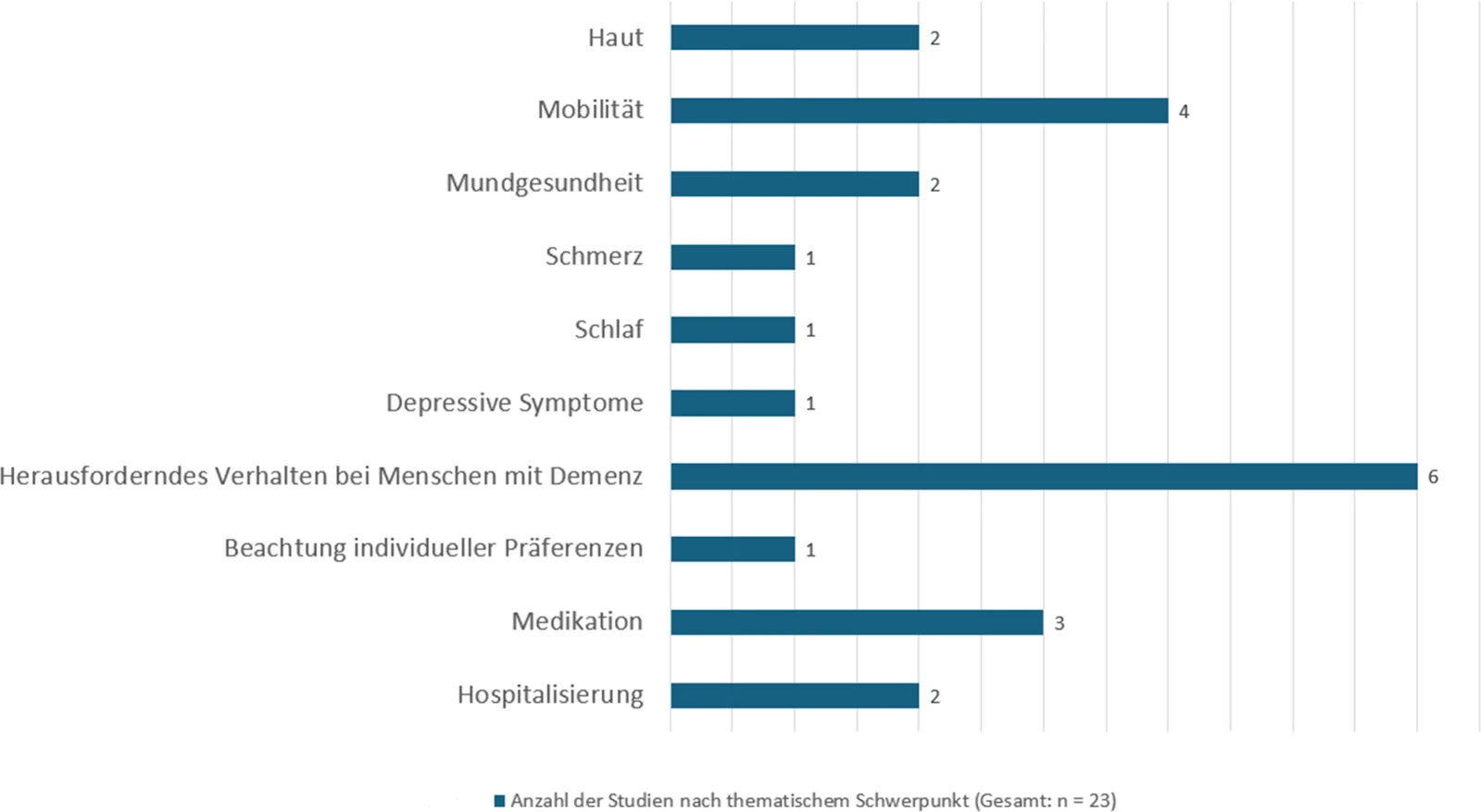
Thematische Schwerpunkte der Interventionsansätze

Bei den Interventionen wurden unterschiedliche thematische Schwerpunkte identifiziert. Die meisten Studien befassten sich mit Mobilitätsaspekten und herausfordernden Verhaltensweisen bei Menschen mit Demenz sowie der Medikation und Hospitalisierung. Die Abb. 3 zeigt einen Überblick über die 10 inhaltlichen Schwerpunkte der Interventionen.

### Haut.

Zwei Studien untersuchten Interventionen, die auf die Verbesserung des Hautzustandes bzw. von Hauterkrankungen abzielten. Eine Studie legte den Fokus ausschließlich auf Hauttrockenheit und untersuchte, inwiefern sich ein strukturierter Behandlungsplan zur Hautpflege darauf auswirkt [64]. Eine weitere Studie betrachtete verschiedene Hauterkrankungen wie Hauttrockenheit und IAD und überprüfte den Einfluss eines Hautpflegeprogramms auf die Prävention und Behandlung dieser Hauterkrankungen [65].

### Mobilität.

Insgesamt 4 Studien befassten sich mit der Mobilität, wobei sie sich hinsichtlich ihrer inhaltlichen Schwerpunktsetzung unterschieden. Eine Studie untersuchte 2 unterschiedliche Versionen eines komplexen Interventionsprogramms zur Vermeidung von freiheitsentziehenden Maßnahmen, die die Mobilität und Fortbewegungsmöglichkeiten der Bewohner:innen einschränken [66]. Cordes et al. [67] fokussierten auf die Förderung der Mobilität von Bewohner:innen, die nicht gehfähig sind, durch eine Bewegungsintervention. Görres et al. [68] evaluierten die Implementierung eines Expertenstandards zur Erhaltung und Förderung der Mobilität. Bei Nguyen et al. [69] wurde eine komplexe Intervention zur Verbesserung von Aktivität und Teilhabe bei Bewohner:innen mit Gelenkkontrakturen untersucht.

### Mundgesundheit.

Zwei Studien untersuchten Interventionen zur Verbesserung der Mundgesundheit, wobei eine Studie spezifisch Teilnehmende mit kognitiven und motorischen Einschränkungen eingeschlossen hat [70]. Im Fokus der Studie von Barbe et al. [71] stand die Durchführung der Mund- und Zahnpflege durch eine zahnmedizinische Fachangestellte, Kern der Intervention bei Zenthöfer et al. [70] war eine Schulung der Pflegenden.

### Schmerz.

In einer Studie wurde die Auswirkung einer Intervention zur Optimierung des Schmerzmanagements auf die Schmerzintensität und die schmerzbedingten Beeinträchtigungen der Funktionsfähigkeit der Teilnehmenden überprüft [72].

### Schlaf.

Mit dem Thema Schlaf befasste sich eine Studie. Hierbei wurde eine komplexe, nichtpharmakologische Intervention entwickelt und evaluiert, um Schlafstörungen bei Menschen mit Demenz zu reduzieren [73].

### Depressive Symptome.

Werner et al. [74] haben sich mit depressiven Symptomen bei Bewohner:innen beschäftigt. Diese sollten durch interaktive Musiktherapie und gemeinschaftliches Singen verringert werden.

### Herausfordernde Verhaltensweisen bei Menschen mit Demenz.

Der Einfluss verschiedener Interventionen auf herausfordernde Verhaltensweisen bei Menschen mit Demenz wurde in 6 Arbeiten untersucht. Zwei Publikationen davon fokussierten auf verschiedene musiktherapeutische Ansätze [75] bzw. individualisiertes Musikhören [76], während O’Sullivan et al. [77] den Einfluss einer Tablet-basierten Intervention zur Verringerung von Apathie untersuchten. Eine weitere Studie beschäftigte sich mit Demenz-spezifischen Fallkonferenzen und untersuchte den Effekt auf die Prävalenz von herausfordernden Verhaltensweisen [78]. Die komplexe, nichtpharmakologische Intervention der Studie von Kratzer et al. [79] zielte neben der Reduktion von herausforderndem Verhalten darauf ab, die Lebensqualität von Menschen mit einer schweren Demenz zu verbessern. Middelstädt et al. [80] untersuchten den Effekt von kognitiver Stimulation u. a. auf herausforderndes Verhalten, Kognition und Lebensqualität.

### Individuelle Versorgungspräferenzen.

Götze et al. [81] untersuchten, inwiefern sich die Implementierung eines Programms zu Advance Care Planning (vorausschauende Versorgungsplanung) auf die Bekanntheit und Einhaltung der Versorgungspräferenzen der Bewohner:innen auswirkt und sich somit die Versorgung danach ausrichtet.

### Medikation.

Hinsichtlich der Medikation der Bewohner:innen wurden verschiedene Aspekte beleuchtet. Eine Studie untersuchte, ob eine komplexe, multiprofessionelle Intervention die Arzneimittelsicherheit verbessert, u. a. durch eine Reduktion von PIM oder Neuroleptika [82]. In einer weiteren Studie wurde der Einfluss einer Überprüfung der Medikation durch Apotheker:innen auf Medikationsänderungen und weitere patientenrelevante Outcomes, wie die Hospitalisierungsrate oder Anzahl an Stürzen, betrachtet [83]. Eine Studie adaptierte eine Intervention zu Person-zentrierter Versorgung auf die deutschen Rahmenbedingungen und prüfte deren Einfluss auf den Anteil von Bewohner:innen mit Antipsychotika-Verordnungen [84].

### Hospitalisierung.

Zum Thema Hospitalisierung wurden 2 Studien eingeschlossen. Beide Studien untersuchten einen Ansatz der interprofessionellen Zusammenarbeit. Während die Arbeit von Mazur et al. [85] den Einfluss von interprofessioneller Zusammenarbeit zwischen Hausärzt:innen und Pflegenden der Einrichtungen auf die Hospitalisierungsrate untersuchte, wurde in der Arbeit von Tönnies et al. [86] der Effekt eines interprofessionellen Versorgungskonzepts, u. a. durch den Einsatz regionaler hausärztlicher Versorgungsteams, auf die Anzahl der Krankenhauseinweisungen untersucht.

## Versorgungsqualität in der stationären Langzeitpflege

Insgesamt ist die Berichterstattung zur Versorgungsqualität in den Pflegeheimen sehr begrenzt. Im Zeitraum der letzten fünf Jahre sind 3 Qualitätsberichte durch den Medizinischen Dienst Bund erschienen [87–89]. Darüber hinaus gibt es einen Pflege-Report aus dem Jahr 2023 [90] mit diesem Schwerpunktthema.

Zentrale Aspekte der Qualitätssicherung von Pflegeeinrichtungen sind im Elften Sozialgesetzbuch (SGB XI) gesetzlich verankert. Die externe Qualitätsprüfung erfolgt dabei durch den Medizinischen Dienst bzw. den Prüfdienst der privaten Krankenversicherung (PKV) auf Grundlage der Qualitätsprüfungs-Richtlinien für die vollstationäre Pflege (QPR). Die Pflegedokumentation, die Inaugenscheinnahme einer Stichprobe der Bewohner:innen und Fachgespräche mit den Pflegenden dienen u. a. als zentrale Datengrundlage zur Beurteilung der Versorgungsqualität. Die Ergebnisse daraus werden in den Prüfberichten des Medizinischen Dienstes Bund veröffentlicht [87–89].

Die Qualitätsberichte des Medizinischen Dienstes Bund erscheinen im Turnus von zwei Jahren und berichten jeweils über die Ergebnisse der Prüfungen aus dem vergangenen Berichtszeitraum. Der 8. Prüfbericht aus dem Jahr 2025 [87] ist der aktuellste Bericht und fasst die Ergebnisse der Qualitätsprüfungen aus dem Jahr 2023 zusammen. Im Zuge eines verlängerten Prüfintervalls in diesem Zeitraum blieben 475 vollstationäre Einrichtungen mit guter Qualität unberücksichtigt, was sich potenziell negativ auf die ermittelten Durchschnittswerte ausgewirkt haben kann. Die vorliegenden Ergebnisse basieren somit lediglich auf 77,6 % der zugelassenen Pflegeheime und erlauben keine uneingeschränkte Übertragbarkeit auf alle Einrichtungen [87]. In den Prüfungen werden verschiedene Qualitätsbereiche betrachtet wie die „Unterstützung bei der Mobilität und die Selbstversorgung“ oder auch die „Unterstützung bei der Bewältigung von krankheits- und therapiebedingten Anforderungen und Belastungen“ [87–89]. Der 7. Prüfbericht aus dem Jahr 2024 basiert auf Daten aus 2021 und war insbesondere beeinflusst durch die Herausforderungen im Zusammenhang mit der COVID-19-Pandemie, in deren Verlauf die jährlichen Regelprüfungen zeitweise ausgesetzt wurden. Somit konnte in diesem Zeitraum lediglich in 6692 (59 %) vollstationären Einrichtungen eine externen Qualitätsprüfung umgesetzt werden [88]. Der im Jahr 2021 erschienene 6. Pflege-Qualitätsbericht war der erste Prüfbericht, in den Daten aus Qualitätsprüfungen nach dem im Jahr 2019 eingeführten neuen Prüfverfahren für die stationäre Pflege eingeflossen sind. Zudem fanden hier bereits aufgrund der COVID-19-Pandemie zeitweise nur wenige Qualitätsprüfungen statt [89].

Die jährlich erscheinenden Pflege-Reports greifen zentrale Entwicklungen und Herausforderungen in der Pflege auf – der Pflege-Report 2023 legt dabei beispielsweise einen besonderen Fokus auf die Versorgungsqualität von Langzeitgepflegten [90]. Ein Schwerpunkt liegt auf dem Qualitätsatlas Pflege, der mithilfe von sogenannten QCare-Indikatoren (Qualitätsindikatoren basierend auf Routinedaten der AOK, mit denen die Versorgungsqualität in Pflegeheimen bewertet und verglichen werden kann) eine raumbezogene Qualitätsmessung bei Pflegeheimbewohner:innen ermöglicht und regionale Unterschiede sichtbar macht. Darüber hinaus wird unter dem Titel „Forschung für mehr Qualität“ ein deskriptiver Überblick über Förderprogramme im Rahmen des SGB V und SGB XI gegeben, die auf eine Weiterentwicklung der pflegerischen Versorgung abzielen. Weitere Beiträge befassen sich mit Instrumenten und Maßnahmen der Qualitätssicherung und -entwicklung sowie mit der öffentlichen Berichterstattung über Qualitätsleistungen. Zudem wird analysiert, welchen Einfluss die politischen und rechtlichen Rahmenbedingungen der Pflegeversicherung auf die Qualität der pflegerischen Versorgung haben. Ein zentraler Themenkomplex betrifft das Verhältnis von Personal und Qualität in der Langzeitpflege. Hier werden u. a. die Bedeutung und Herausforderungen von Weiterbildungsmaßnahmen, Ansätze wie interprofessionelle Zusammenarbeit zur Qualitätsverbesserung sowie die Rolle primärqualifizierender Studiengänge für die zukünftige Fachkräftesicherung und Qualitätsentwicklung diskutiert.

## Diskussion

Es wurde eine Vielzahl von Studien identifiziert, die die gesundheitliche Lage von Pflegeheimbewohner:innen in verschiedenen Themenfeldern untersuchten. Die Anzahl der Studien zur Evaluation von Interventionsansätzen ist hingegen gering, was möglicherweise auf die besonderen Anforderungen zurückzuführen ist, die mit der Durchführung solcher Interventionsstudien in diesem Setting verbunden sind. Bereits in der Vergangenheit wurde kritisiert, dass es an experimentellen Studien in der Pflege mangelt und die dafür notwendigen methodischen Kompetenzen der Forschenden ausgebaut werden müssen [91]. Es zeigte sich eine große Bandbreite an Themen- und Problemfeldern. Die inhaltlichen Schwerpunkte der Studien lagen insbesondere auf Hautgesundheit, Mobilität, herausfordernden Verhaltensweisen bei Menschen mit Demenz, spezifischen Krankheitsbildern sowie Hospitalisierung und Notfallversorgung.

Trotz der vergleichsweise hohen Anzahl an Studien zur Beschreibung der gesundheitlichen Lage und thematischen Bandbreite ist die Forschungslage weiterhin als unzureichend einzuschätzen. So wurden für relevante Themen- und Problemfelder wie Ernährung, Schmerz, Schlaf, *Frailty*, Mundgesundheit oder soziale Teilhabe nur einzelne Studien identifiziert. Aspekte wie Lebensqualität fehlten gänzlich. Die Diskrepanz zwischen den empirischen Studien zur gesundheitlichen Lage und den Studien zu Interventionsansätzen sowie die inhaltliche Schwerpunktsetzung sind möglicherweise auf das Forschungsinteresse in diesem Feld und die Ausrichtung der Forschungsförderung der vergangenen Jahre sowie die oben genannten methodischen Herausforderungen zurückzuführen. Aus den Ergebnissen wird zudem eine hohe Heterogenität in der Berichterstattung der Studien sowie in der Anzahl der eingeschlossenen Teilnehmenden und Einrichtungen deutlich. Es zeigte sich eine hohe Varianz zwischen den berichteten Ergebnismaßen, wodurch die Vergleichbarkeit und Aussagekraft eingeschränkt sind. Diesbezüglich ist auch zu berücksichtigen, dass aufgrund der thematischen Breite im Rahmen dieses narrativen Reviews Informationen wie Erhebungszeiträume oder Erhebungsmethoden und ggf. -instrumente nicht in die Betrachtung einbezogen wurden. Zudem war die Recherche für das narrative Review auf eine wissenschaftliche Datenbank und den Zeitraum der letzten 10 Jahre eingeschränkt und erhebt somit keinen Anspruch auf Vollständigkeit.

Die Berichterstattung zur Versorgungsqualität in den stationären Langzeitpflegeeinrichtungen ist insgesamt begrenzt. Dennoch lässt sich festhalten, dass die Diskussion zur Qualität in Pflegeheimen eng mit den strukturellen Rahmenbedingungen wie der Personalsituation oder einer zunehmenden Komplexität der Versorgung verknüpft ist. Die Verbesserung der Rahmenbedingungen und die Sicherung einer qualitativ hochwertigen Versorgung stellen eine zentrale Zukunftsaufgabe dar und erfordern innovative Konzepte. Beispielhaft adressiert das Projekt *Transfercluster Akademischer Lehrpflegeeinrichtungen in der Langzeitpflege (TCALL)* diese Herausforderungen, indem es (orientiert am internationalen Konzept der „Teaching Nursing Homes“) durch den Aufbau eines akademischen Lehrpflegeeinrichtungsclusters den Transfer zwischen Wissenschaft und Praxis über die Ausbildung stärken möchte und so eine evidenzbasierte Versorgung fördern will [92].

## Fazit

Dieses narrative Review gibt einen Überblick über die empirischen Befunde der letzten 10 Jahre zur gesundheitlichen Lage hochaltriger Menschen in stationären Langzeitpflegeeinrichtungen in Deutschland sowie über entsprechende Interventionsansätze und aktuelle Berichte zur Versorgungsqualität. Zahlreiche Studien beleuchten zwar die gesundheitliche Lage in verschiedenen Themenfeldern, bestimmte relevante Problembereiche werden dabei jedoch nicht oder nur teilweise untersucht. Die Studienlage bei entsprechenden Interventionsansätzen und auch die Berichterstattung zur Versorgungsqualität in den Pflegeheimen sind begrenzt. Insgesamt bleibt daher festzuhalten, dass die gesundheitliche Lebenssituation hochaltriger Menschen in Pflegeeinrichtungen unzureichend erforscht ist und es neben der Entwicklung von wissenschaftlich basierten Interventionsansätzen weiterer methodisch robuster Studien mit angemessenen Erhebungsmethoden und Ergebnismaßen bedarf, um die spezifischen Bedarfslagen dieser Personengruppe umfassend zu erfassen und zu berichten.

## Supplementary Information

Onlinematerial 1: Studienmerkmale zur Gesundheit von Pflegeheimbewohner:innen

Onlinematerial 2: Merkmale der Interventionsstudien

## Data Availability

Alle dieser Arbeit zugrunde liegenden Daten sind in diesem Artikel enthalten.
