# Supplementary material for: Health status of older people in long-term inpatient care—a narrative review
Source: Bundesgesundheitsblatt Gesundheitsforschung Gesundheitsschutz. 2026 Aug 7;69(9):1018–28. [Article in German] doi: 10.1007/s00103-026-04283-x (PMC13562270; doi:10.1007/s00103-026-04283-x)
Supplement: Supplementary file 1 [file 103_2026_4283_MOESM1_ESM.pdf]

# Onlinematerial 1

eTab. 1 Studienmerkmale zur Gesundheit von Pflegeheimbewohner:innen

| Referenz* | Anzahl der Einrichtungen/Cluster | Merkmale der Stichprobe | Thematischer Fokus | Hauptergebnisse |
|---|---|---|---|---|
| Haut (Hautzustand, Hauterkrankungen, chronische und schwerheilende Wunden) | | | | |
| Akdeniz et al. (2019) | n = 10 Pflegeheime | Gesamt: n = 223 Bewohner:innen<br>Alter: 83,6 Jahre (SD: 8,0)<br>Weiblich: 68 % | Vorstufenläsionen und Hautkrebs | **Prävalenz**<br>Androgenetic alopecia: 50,2 %<br>Melanozytäre Nävi: 13 %<br>Aktinische Keratose: 21,1 %<br>Morbus Bowen: 3,1 %<br>Lentigo maligna: 0,4 %<br>Nicht-melanotischer Hautkrebs: 7,2 %<br>Malignes Melanom: 0 % |
| Boronat-Garrido et al. (2016) | n = 78 Pflegeheime | Gesamt: n = 3.406 Bewohner:innen<br>Alter: k. A.<br>Weiblich: k. A. | Inkontinenz-assoziierte Dermatitis | **Prävalenz**<br>5,2 % |
| Gabriel et al. (2019) | n = 10 Pflegeheime | Gesamt: n = 223 Bewohner:innen<br>Alter: 83,6 (SD: 8,0)<br>Weiblich: 68 % | Intertrigo | **Prävalenz**<br>16,1 % |
| Hahnel et al. (2017) | n = 10 Pflegeheime | Gesamt: n = 223 Bewohner:innen<br>Alter: 83,6 % (SD: 8,0)<br>Weiblich: 68 % | Hautbeschaffenheit und Hauterkrankungen | **Prävalenz**<br>Xerosis cutis: 99,1 %<br>Tinea unguium: 62,3 %<br>Seborrhoische Keratose: 56,5 % |

| Referenz* | Anzahl der Einrichtungen/Cluster | Merkmale der Stichprobe | Thematischer Fokus | Hauptergebnisse |
|---|---|---|---|---|
| Lechner et al. (2017) | n = 30 Pflegeheime | Gesamt: n = 2.085 Bewohner:innen<br>Alter: 81,4 Jahre (SD: 12,1)<br>Weiblich: 68 % | Hauttrockenheit und Dekubitus | **Prävalenz**<br>Hauttrockenheit: 45,8 %<br>Dekubitus:<br>Kategorie 1–4 & Tiefengewebeschädigung: 3,4 %<br>Kategorie 2–4 & Tiefengewebeschädigung: 2,1 %<br>Kategorie 2–4 & Tiefengewebeschädigung Kreuzbein/Trochanter: 1,3 %<br>Kategorie 2-4 & Tiefengewebeschädigung Ferse/Knöchel: 0,7 % |
| Lichtenfeld et al. (2016) | *nicht berichtet* | Gesamt: n = 1.091 Bewohner:innen<br>Alter: 81,6 Jahre (SD: 12,3)<br>Weiblich: 69 % | Hauttrockenheit | **Prävalenz**<br>Hauttrockenheit: 52,6 % |
| Lichtenfeld-Kottner et al. (2020) | *nicht berichtet* | Gesamt: n = 38.655 Bewohner:innen<br>Alter: 85,4 Jahre (SD: 9,4)<br>Weiblich: 77 % | Im institutionellen Bereich erworbene Druckgeschwüre | **Prävalenz**<br>Im institutionellen Bereich erworbene Druckgeschwüre (Grad I-IV):<br>*Keine Angabe Gesamt*<br>Frauen: 3,3 % (n = 987)<br>Männer: 3,3 % (n = 278) |
| Raeder et al. (2020) | *nicht berichtet* | Gesamt: n = 7.662 Bewohner:innen<br>Alter: 85 Jahre (SD: k. A.)<br>Weiblich: k. A. | Chronische Wunden | **Prävalenz**<br>7,8 % |

| Referenz* | Anzahl der Einrichtungen/Cluster | Merkmale der Stichprobe | Thematischer Fokus | Hauptergebnisse |
|---|---|---|---|---|
| Völzer et al. (2023) | n = 17 Pflegeheime | Gesamt: n = 314 Bewohner:innen<br>Alter: 85,4 Jahre (SD: 7,1)<br>Weiblich: 69 % | Hautzustand | **Prävalenz**<br>Xerosis cutis: 95,9 %<br>Intertrigo: 35,0 %<br>Inkontinenzassoziierte Dermatitis: 21,0 % (n = 52/248)<br>Hautrisse: 10,5 %<br>Druckgeschwüre 8,0 % |
| Ernährung | | | | |
| Streicher et al. (2017) | *Nicht berichtet* | Gesamt: n = 3.325 Bewohner:innen<br>Alter: k. A.<br>Weiblich: k. A. | Orale Nahrungsergänzungsmittel | **Prävalenz**<br>Anteil der Bewohner:innen, die orale Nahrungsergänzungsmittel erhalten: 8,3 % |
| Strube-Lahrmann et al. (2021) | n = 280 Pflegeheime | Gesamt: n = 19.686 Bewohner:innen<br>Alter: 85,9 Jahre (SD: 9,2) (Frauen)<br>78,6 Jahre (SD: 12,9) (Männer)<br>Weiblich: 75 % | Untergewicht | **Prävalenz**<br>Anteil der Bewohner:innen mit Untergewicht: 13,7 % |
| Schmerz | | | | |
| Budnick et al. (2020) | n = 12 Pflegeheime | Gesamt: n = 137 Bewohner:innen<br>Alter: 83,3 Jahre (SD: k. A.)<br>Weiblich: 72 % | Chronische Schmerzen | **Prävalenz**<br>Gelinderte Schmerzen: 46,7 %<br>Niedrig bis moderate Schmerzen: 22,6 %<br>Starke Schmerzen: 30,7 % |

| Referenz* | Anzahl der Einrichtungen/Cluster | Merkmale der Stichprobe | Thematischer Fokus | Hauptergebnisse |
|---|---|---|---|---|
| Centmayer et al. (2024) | n = 51 Pflegeheime | Gesamt: 3.482 Bewohner:innen<br>Alter: 81,7 Jahre (SD: 11,9)<br>Weiblich: 71 % | Schmerzprävalenz und Schmerzmanagement bei Bewohner:innen mit und ohne kognitiven Einschränkungen | **Prävalenz**<br>Bewohner:innen mit Schmerzen: 31,8 %<br><br>Davon:<br>Keine kognitive Einschränkung: 40,4 %<br>Moderate kognitive Einschränkung: 34,0 %<br>Schwere kognitive Einschränkung: 24,9 % |
| Wróblewska et al. (2019) | *Nicht berichtet* | Gesamt: n = 190 Bewohner:innen<br>Alter: 89 Jahre (SD: 7,2)<br>Weiblich: 100 % *(männliche Teilnehmende wurden nicht eingeschlossen)* | Schmerz und Symptome von Depression | **Prävalenz**<br>Rückenschmerz: 63 %<br>Schwindel: 53 %<br>Schlafstörungen: 48 %<br>Subjektive Einschätzung von körperlichem Schmerz als mittel: 71 %/schlecht: 10 %<br>Generelle Einschätzung der Gesundheit als schlecht: 45 % |
| Schlaf | | | | |
| Wilfing et al. (2019) | n = 38 Pflegeheime | Gesamt: n = 1.187 Bewohner:innen<br>Alter: 83 Jahre (SD: 9,4)<br>Weiblich: 74 % | Schlafstörungen bei Bewohner:innen mit Demenz | **Prävalenz**<br>Anteil der Bewohner:innen mit Schlafstörungen: 23 % (Range: 0-85 %) |
| Kontinenz | | | | |
| Czwikla et al. (2024) | n = 21 Pflegeheime | Gesamt: n = 852 Bewohner:innen<br>Alter: 83,5 Jahre (SD: 10,5)<br>Weiblich: 77 % | Einsatz von Blasendauerkathetern | **Prävalenz**<br>13,4 % |

| Referenz* | Anzahl der Einrichtungen/Cluster | Merkmale der Stichprobe | Thematischer Fokus | Hauptergebnisse |
|---|---|---|---|---|
| Soziale Teilhabe | | | | |
| Gebhard et al. (2024) | n = 12 Pflegeheime | Gesamt: n = 106 Bewohner:innen<br>Alter: 85,2 Jahre (SD: 7,4)<br>Weiblich: 83 % | Soziale Interaktionen von Menschen mit Demenz | **Anteil der Bewohner:innen**<br>Gesamt: n = 6.134 Beobachtungen<br>Keine soziale Interaktion: 71,9 %<br>Mit Mitarbeitenden: 43,4 %<br>Mit anderen Bewohner:innen: 40,9 %<br>Mit Familie/Freund:innen: 3,9 %<br>Andere: 11,7 % |
| Hajek et al. (2024) | *nicht berichtet* | Gesamt: n = 142 Bewohner:innen<br>Alter: 90,2 Jahre (SD: 4,9)<br>Weiblich: 73 % | Einsamkeit | **Prävalenz**<br>Nicht einsam: 56,6 %<br>Moderat einsam: 25,7 %<br>Stark einsam: 17,8 % |
| Herausforderndes Verhalten bei Menschen mit Demenz | | | | |
| Hüsken et al. (2024) | n = 21 Pflegeheime | Gesamt: n = 699 Bewohner:innen (mit Demenz)<br>Alter: 84,1 Jahre (SD: 7,8)<br>Weiblich: 75 % | Neuropsychiatrische Symptome bei Menschen mit Demenz | **Prävalenz**<br>Erregung/Aggression: 36 %<br>Depression/Dysphorie: 33 %<br>Apathie/Gleichgültigkeit: 33 %<br>Reizbarkeit/Labilität: 30 %<br>Abweichendes motorisches Verhalten: 27 %<br>Verhalten in der Nacht: 25 %<br>Angst: 23 %<br>Veränderung des Appetits und des Essverhaltens: 23 %<br>Enthemmung: 16 %<br>Wahnvorstellung: 14 %<br>Halluzination: 9 %<br>Euphorie/Hochstimmung: 6 % |

| Referenz* | Anzahl der Einrichtungen/Cluster | Merkmale der Stichprobe | Thematischer Fokus | Hauptergebnisse |
|---|---|---|---|---|
| Palm et al. (2018) | *Anzahl der Pflegeheime nicht berichtet*<br>n = 140 Wohnbereiche | Gesamt: n = 1.967 Bewohner:innen<br>Alter: 83,4 Jahre (SD: 8,0)<br>Weiblich: 77 % | Agitiertes Verhalten bei Menschen mit Demenz | **Prävalenz**<br>6,3 % |
| Einschränkungen der Sinneswahrnehmung | | | | |
| Bock et al. (2025) | n = 4 Pflegeheime | Gesamt: n = 102 Teilnehmende<br>Alter: 82,6 Jahre (SD: 10,0)<br>Weiblich: k. A. | Hörsituation und Hörversorgung | **Prävalenz**<br>Hörgeräteindikation ja, davon<br>Hörgerätbesitzende: 45,5 %<br>Personen ohne Hörgerät: 28,8 %<br><br>Hörgeräteindikation nein, davon<br>Hörgerätbesitzende: 4,5 %<br>Personen ohne Hörgerät: 21,2 %<br><br>Personen ohne Hörgerät, aber mit wahrgenommenem Hörverlust: 18,6 % |
| Larsen et al. (2019) | n = 32 Pflegeheime | Gesamt: n = 566 Bewohner:innen<br>Alter: 82,9 Jahre (SD: 9,8)<br>Weiblich: 73 % | Sehbeeinträchtigungen | **Prävalenz**<br>Moderate Sehbeeinträchtigung: 16,8 %<br>Schwere Sehbeeinträchtigung: 1,9 %<br>Erblindung: 5,3 % |

| Referenz* | Anzahl der Einrichtungen/Cluster | Merkmale der Stichprobe | Thematischer Fokus | Hauptergebnisse |
|---|---|---|---|---|
| Thederan et al. (2016) | n = 17 Pflegeheime | Gesamt: n = 314 Bewohner:innen<br>Alter: 85,4 Jahre (SD: 7,1)<br>Weiblich: n = 119 | Häufigkeit von Augenerkrankungen | **Prävalenz**<br>Akuter ophthalmologischer Behandlungsbedarf: 21,7 %<br>Häufigste Diagnosen im vorderen Augenabschnitt:<br>Keratokonjunktivitis sicca (78,8 %), Katarakt (43,3 %), sekundärer Katarakt (7,4 %), Glaukom (12,3 %) und Fehlstellungen der Augenlider (12,3 %).<br>häufigsten Diagnosen im Fundus: trockene altersbedingte Makuladegeneration (AMD) (22,2 %), frische feuchte AMD (3,4 %) und epiretinale Gliose (3,4 %)<br>39,9 % der Bewohner:innen konnten keine Angaben zu früheren Augenbehandlungen machen |
| Mundgesundheit | | | | |
| Klotz et al. (2017) | n = 14 Pflegeheime | Gesamt: n = 169 Bewohner:innen<br>Alter: 82,9 Jahre (SD: 8,9)<br>Weiblich: 67 % | Mundgesundheitsbezogene Lebensqualität | **Anteil der Bewohner:innen**<br>Beeinträchtigte mundgesundheitsbezogene Lebensqualität: 41 % |
| Medikation | | | | |
| Allers et al. (2017) | n = 21 Pflegeheime | Gesamt: n = 837 Bewohner:innen<br>Alter: 83,4 Jahre (SD: 10,5)<br>Weiblich: 76 % | Antipsychotika bei Pflegeheimbewohner:innen mit und ohne Demenz | **Prävalenz**<br>Insgesamt: 35,4 %<br>Personen mit Demenz: 47,0 %<br>Personen ohne Demenz: 19,5 % |
| Ezzat et al. (2019) | nicht berichtet | Gesamt: n = 188 Bewohner:innen<br>Alter: 80,7 Jahre (SD: k. A.)<br>Weiblich: 73 % | Verordnungshäufigkeit von Psychopharmaka mit sedierender und potenziell freiheitseinschränkender Wirkung | **Prävalenz**<br>Mind. eine Regelverordnung: 55,3 %<br>Sedierendes Regelmedikament: 33,5 % |

| Referenz* | Anzahl der Einrichtungen/Cluster | Merkmale der Stichprobe | Thematischer Fokus | Hauptergebnisse |
|---|---|---|---|---|
| Hoffmann & Schmiemann (2016) | n = 21 Pflegeheime | Gesamt: n = 852 Bewohner:innen<br>Alter: 83,5 Jahre (SD: 10,5)<br>Weiblich: 77 % | Verschreibung von Schmerzmedikation | **Prävalenz**<br>Mindestens ein Analgetikum: 73,8 % |
| Lexow et al. (2021) | n = 3 Pflegeheime | Gesamt: n = 104 Bewohner:innen<br>Alter: 86 Jahre (SD: k. A.)<br>Weiblich: 72 % | Adverse Effekte im Zusammenhang mit Polypharmazie | **Prävalenz**<br>Bewohner:innen mit Adversen Effekten: 99 % Adverse Effekte gesamt: (Median: 4 pro Bewohner:in; Range 1–14) |
| Lukačišinová et al. (2021) | n = 9 Pflegeheime | *Keine Charakteristika berichtet* | Häufigkeit von Benzodiazepinen und Z-Medikamenten | **Prävalenz**<br>Bewohner:innen mit Benzodiazepinen/Z-Medikamenten: 14,5 % |
| Makan et al. (2021) | *nicht berichtet* | Gesamt: n = 468 Bewohner:innen<br>Alter: k. A.<br>Weiblich: k. A. | Pharmakologische Osteoporoseprävention (Vitamin D, Kalzium, Bisphosphonate) | **Prävalenz**<br>Bewohner:innen mit mind. einem Medikament: 8,3 % |
| Makan et al. (2024) | *nicht berichtet* | Gesamt: n = 468 Bewohner:innen<br>Alter: k. A.<br>Weiblich: k. A. | Potenziell inadäquate Medikamente (PIM) | **Prävalenz**<br>Bewohner:innen mit mind. einem PIM: 84,4 % |
| Makan et al. (2017) | *nicht berichtet* | Gesamt: n = 464 Bewohner:innen<br>Alter: k. A.<br>Weiblich: k. A. | Präventive Einnahme von Medikamenten gegen kardiovaskuläre Erkrankungen | **Prävalenz**<br>Bewohner:innen mit mind. einem Medikament: 64,7 % |

| Referenz* | Anzahl der Einrichtungen/Cluster | Merkmale der Stichprobe | Thematischer Fokus | Hauptergebnisse |
|---|---|---|---|---|
| Rausch et al. (2020) | *nicht berichtet (Versichertendaten als Grundlage)* | Bewohner:innen mit Demenz:<br>Gesamt: n =29.052 Bewohner:innen<br>Alter: 86,4 Jahre (SD: 6,5)<br>Weiblich: 69 %<br>Bewohner:innen ohne Demenz:<br>Gesamt: n = 38.276 Bewohner:innen<br>Alter: 84,6 Jahre (SD: 7,9)<br>Weiblich: 71 % | Verordnung von Medikamenten mit fraglichem Nutzen in den letzten 6 Lebensmonaten bei älteren Bewohner:innen mit und ohne Demenz | **Prävalenz**<br>Mind. 1 Medikament mit fraglichem Nutzen:<br>Bewohner:innen mit Demenz:<br>180–91 Tage vor dem Versterben (29.6 %)<br>90 Tage vor dem Versterben (26.8 %)<br>Bewohner:innen ohne Demenz:<br>180–91 Tage vor dem Versterben (22.8 %)<br>90 Tage vor dem Versterben (20.1%) |
| Weinand et al. (2022) | *nicht berichtet (Versichertendaten als Grundlage)* | Gesamt: n = 259.328 Bewohner:innen<br>Alter: 85,8 Jahre (SD: k. A.)<br>Weiblich: 74 % | Potenziell inadäquate Medikation (PIM) | **Prävalenz**<br>Bewohner:innen mit mind. einer PIM: 25,5 % |
| Spezifische Diagnosen/Gesundheitsbeeinträchtigungen/Syndrome | | | | |
| Fassmer et al. (2018) | nicht berichtet (Versichertendaten als Grundlage) | Gesamt: n = 127.227 Bewohner:innen<br>Alter: 84 Jahre (SD: 7,2)<br>Weiblich: 74 % | Pneumonie | **Inzidenzrate pro 100 Personenjahre**<br>11,8 |
| Grosshauser et al. (2023) | n = 7 Pflegeheime | Gesamt: n = 246 Bewohner:innen<br>Alter: 83,6 Jahre (SD: 8,3)<br>Weiblich: 67 % | Frailty | **Prävalenz in %**<br>**FRAIL-NH Scale**<br>Sehr gebrechlich: 71,1 %<br>Gebrechlich: 26,4 %<br>Nicht gebrechlich: 2,5 %<br>**Clinical Frailty Scale (CFS)**<br>Schwer gebrechlich: 66,3 %<br>Leicht bis mäßig gebrechlich: 26,8 %<br>Nicht gebrechlich: 6,9 % |

| Referenz* | Anzahl der Einrichtungen/Cluster | Merkmale der Stichprobe | Thematischer Fokus | Hauptergebnisse |
| --- | --- | --- | --- | --- |
| Haigis et al. (2024) | n = 5 Pflegeheime | Gesamt: n = 63 Bewohner:innen<br><br>Keine Sarkopenie<br>Gesamt: n = 27 Bewohner:innen<br>Alter: 86 Jahre (SD: k. A.)<br>Weiblich: 77 %<br>Mögliche Sarkopenie<br>Gesamt: n = 26 Bewohner:innen<br>Alter: 86 Jahre (SD: k. A.)<br>Weiblich: 65 %<br>Bestätigte/schwere Sarkopenie<br>Gesamt: n = 10 Bewohner:innen<br>Alter: 89 Jahre (SD: k. A.)<br>Weiblich: 90 % | Sedentäres Verhalten und Sarkopenie | **Prävalenz**<br>Keine Sarkopenie: 42,9 %<br>Mögliche Sarkopenie: 41,2 %<br>Bestätigte/schwere Sarkopenie: 15,9 % |
| Hoffmann et al. (2016) | n = 21 Pflegeheime | Gesamt: n = 852 Bewohner:innen<br>Alter: 83,5 Jahre (SD: 10,5)<br>Weiblich: 76 % | Häufigkeit von Niereninsuffizienz | **Prävalenz**<br>63,6 % |
| Mai et al. (2022) | n = 57 Pflegeheime<br>n = 85 Wohnbereiche | Gesamt: n = 1.418 Bewohner:innen<br>Alter: k. A.<br>Weiblich: k. A. | Häufigkeit von Parkinson | **Prävalenz**<br>Bewohner:innen mit Parkinson: 13,9 % |

| Referenz* | Anzahl der Einrichtungen/Cluster | Merkmale der Stichprobe | Thematischer Fokus | Hauptergebnisse |
|---|---|---|---|---|
| Streicher et al. (2018) | *Nicht berichtet* | Gesamt: n = 3.544 Bewohner:innen<br>Alter: k. A.<br>Weiblich: k. A. | Häufigkeit von Dysphagie | **Prävalenz**<br>Anteil der Bewohner:innen mit Dysphagie: 12 % |
| Zhang et al. (2018) | *nicht berichtet (Versichertendaten als Grundlage)* | Bewohner:innen mit MS:<br>Gesamt: n = 5.692 Bewohner:innen<br>Alter: 76,5 Jahre (SD: 7,8)<br>Weiblich: 74 %<br>Bewohner:innen ohne MS:<br>Gesamt: n = 28.460 Bewohner:innen<br>Alter: 76,5 Jahre (SD: 7,8)<br>Weiblich: 74 % | Hüftfrakturen bei Bewohner:innen mit Multipler Sklerose (MS) | **Adjust. Inzidenzrate**<br>Bewohner:innen mit MS: 7,1 pro 1.000 Personenjahre<br>Bewohner:innen ohne MS: 18,6 pro 1.000 Personenjahre |
| (Fach-)ärztliche Versorgung | | | | |
| Czwikla et al. (2023) | *nicht berichtet* | Gesamt: n = 21.167 Bewohner:innen<br>Alter: 84,9 Jahre (SD: 7,6)<br>Weiblich: 69 % | Inanspruchnahme zahnmedizinischer Leistungen | **Anteil der Bewohner:innen**<br>53,1 % |

| Referenz* | Anzahl der Einrichtungen/Cluster | Merkmale der Stichprobe | Thematischer Fokus | Hauptergebnisse |
|---|---|---|---|---|
| Czwikla et al. (2022) | n = 43 Pflegeheime | Gesamt: n = 400 Bewohnerinnen<br>Alter: 83 Jahre (SD: 9,4)<br>Weiblich: 67 % | Inanspruchnahme hausärztlicher, zahnärztlicher und weiterer fachärztlicher Leistungen | **Anteil der Bewohner:innen (mind. ein Kontakt in den letzten 12 Monaten)**<br>Allgemeinmedizin: 97,8 %<br>Zahnmedizin: 32,3 %<br>Urologie: 18,0 %<br>Neurologie/Psychiatrie: 38,5 %<br>Hals-Nasen-Ohren-Heilkunde: 21,8 %<br>Augenheilkunde: 17,3 %<br>Chirurgie: 6,8 %<br>Dermatologie: 17,0 %<br>Gynäkologie: 3,0 % (n = 267 weibliche Bewohner:innen)<br>Orthopädie: 5,5 % |
| Fang et al. (2017) | n = 32 Pflegeheime | Gesamt: n = 600 Bewohner:innen<br>Alter: 82,9 Jahre (SD: 9,8)<br>Weiblich: 72 % | Ophthalmologische Versorgung im Pflegeheim | **Prävalenz**<br>Subjektiv berichtete Sehprobleme: 47 %<br>behandlungsbedürftige ophthalmologische Befunde: 61 % |
| Schiemann et al. (2016) | n = 21 Pflegeheime | Gesamt: n = 852 Bewohner:innen<br>Alter: 83,5 Jahre (SD: 6,5)<br>Weiblich: 77 % | Versorgung durch Haus- und Fachärzt:innen | **Prävalenz**<br>Ärztlicher kontakt innerhalb der letzten 12 Monate:<br>*Keine Angabe Gesamt*<br>Hausärztlicher Kontakt: ca. 100 %<br>Augenärztlicher Kontakt: 29 %<br>Zahnärztlicher Kontakt: 46 %<br>Kontakt Neurologin/Neurologen oder Psychiater:in: 52 % |

| Referenz* | Anzahl der Einrichtungen/Cluster | Merkmale der Stichprobe | Thematischer Fokus | Hauptergebnisse |
|---|---|---|---|---|
| Schulz et al. (2021) | n = 44 Pflegeheime | Gesamt: n = 409 Bewohner:innen<br>Alter: k. A.<br>Weiblich: 67 % | Fachärztliche Unterversorgung für die Versorgungsbereiche Sehfähigkeit, Hörfähigkeit, Mundgesundheit und Parkinson-Syndrom | **Anteil der Bewohner:innen**<br>Sehfähigkeit: 45 %<br>Hörfähigkeit: 15 %<br>Mundgesundheit: 16 %<br>Parkinson-Syndrom: 19 % |
| Hospitalisierung/Notfallversorgung | | | | |
| Allers et al. (2018) | *nicht berichtet (Versichertendaten als Grundlage)* | Gesamt: n = 127.227 Bewohner:innen<br>Alter: 84,0 (SD: 7,2)<br>Weiblich: 75 % | Mortalität und Hospitalisierung von neuaufgenommenen Bewohner:innen mit und ohne Demenz | **Mortalitätsrate (pro 100 Personenjahre)**<br>Gesamt: 37,9<br>Mit Demenz: 34,6<br>Ohne Demenz: 40,8<br>**Mediane Überlebenszeit (in Tagen)**<br>Gesamt: 706 Tage<br>Mit Demenz: 777 Tage<br>Ohne Demenz: 635 Tage<br>**Anteil der Bewohner*innen mit Hospitalisierung (im letzten Lebensmonat)**<br>Gesamt: 51,5 %<br>Mit Demenz: 51,3 %<br>Ohne Demenz: 51,6 % |
| Fassmer et al. (2020a) | *nicht berichtet (Versichertendaten als Grundlage)* | Gesamt: n = 1.665 Bewohner:innen<br>Alter: 80,5 Jahre (SD: 11,0)<br>Weiblich: 66 % | Inanspruchnahme akuter medizinischer Versorgung (ärztlicher Versorgung außerhalb der Sprechzeiten, Notaufnahmen (mit Entlassung) und akute Krankenhauseinweisungen) | **Inzidenzrate pro Personenjahr**<br>Gesamt: 2,7<br>Ärztliche Versorgung außerhalb der Sprechzeiten: 1,0<br>Notaufnahme: 0,5<br>Akute Krankenhauseinweisung: 1,2 |

| Referenz* | Anzahl der Einrichtungen/Cluster | Merkmale der Stichprobe | Thematischer Fokus | Hauptergebnisse |
|---|---|---|---|---|
| Fassmer et al. (2020b) | n = 14 Pflegeheime | Gesamt: n = 802 Bewohner:innen<br>Alter: 83,8 Jahre (SD: 9,3)<br>Weiblich: 70 % | Geschlechterspezifische Unterschiede bei ungeplanten Krankenhauseinweisungen | **Absolute Häufigkeiten (ungeplante Transporte in einem Jahr)**<br>Gesamt: n = 535<br>Weiblich: n = 375<br>Männlich: n = 159 |
| Hoffmann et al. (2020) | *nicht berichtet (Versichertendaten als Grundlage)* | Gesamt: n = 10.781 Bewohner:innen<br>Alter: 86,1 Jahre (SD: 7,9)<br>Weiblich: 72 % | Hospitalisierung am Lebensende (10-Jahres Trend) | **Anteil der Bewohner:innen**<br>Todestag: 29,2 %<br>Letzte 3 Tage: 31,6 %<br>Letzte 7 Tage: 35,7 %<br>Letzte 14 Tage: 41,2 %<br>Letzte 30 Tage: 48,6 % |
| Hoffmann et al. (2021) | *nicht berichtet (Versichertendaten als Grundlage)* | Gesamt: n = 67.328 Bewohner:innen<br>Alter: 85,3 Jahre (SD: k. A.)<br>Weiblich: 70 % | Hospitalisierung in der letzten Lebensphase | **Anteil der Bewohner:innen**<br>0 Tage vor dem Tod: 29,5 %<br>3 Tage vor dem Tod: 32,4 %<br>7 Tage vor dem Tod: 37,2 %<br>14 Tage vor dem Tod: 43,6 %<br>28 Tage vor dem Tod: 51,5 %<br>90 Tage vor dem Tod: 63,9 %<br>180 Tage vor dem Tod: 69,6 %<br>365 Tage vor dem Tod: 74,3 % |
| Leutgeb et al. (2019) | *nicht berichtet (Versichertendaten als Grundlage)* | Gesamt: n = 13.478 Bewohner:innen<br>Alter: 80,6 Jahre (SD: 13,31)<br>Weiblich: 69 % | Potenziell vermeidbare Hospitalisierungen | **Prävalenz**<br>Anzahl von Krankenhausaufnahmen: n = 23 982<br>Davon 26,9 % (n = 6.449) potenziell vermeidbare Hospitalisierungen |

| Referenz* | Anzahl der Einrichtungen/Cluster | Merkmale der Stichprobe | Thematischer Fokus | Hauptergebnisse |
|---|---|---|---|---|
| Lienesch et al. (2025) | n = 44 Pflegeheime | Gesamt: n = 38.655 Bewohner:innen<br>Altersgruppen:<br>60–74 Jahre: 18,7 %<br>75–84 Jahre: 33,5 %<br>85+ Jahre: 47,8 %<br>Weiblich: 67 % | Krankenhausaufenthalte, Inanspruchnahme medizinischer Notfallversorgung und Kontakte zu den regionalen ärztlichen Bereitschaftsdiensten | **Prävalenz (mind. einmal in den letzten 12 Monaten)**<br>Hospitalisierungen: 45,8 %<br>Inanspruchnahme Notfallversorgung: 23,2 %<br>Kontakt ärztl. Bereitschaftsdienst: 12,1 % |

*Referenzliste findet sich im Hauptmanuskript

Abkürzungen: AMD = altersbedingte Makuladegeneration, k. A. = keine Angabe, SD = Standardabweichung, MS = Multiple Sklerose, PIM = potenziell inadäquate Medikamente
