# Supplementary material for: Health status of older people in long-term inpatient care—a narrative review
Source: Bundesgesundheitsblatt Gesundheitsforschung Gesundheitsschutz. 2026 Aug 7;69(9):1018–28. [Article in German] doi: 10.1007/s00103-026-04283-x (PMC13562270; doi:10.1007/s00103-026-04283-x)
Supplement: Supplementary file 2 [file 103_2026_4283_MOESM2_ESM.pdf]

Onlinematerial 2

eTab. 2 Merkmale der Interventionsstudien

Referenz*	Anzahl der Einrichtungen	Merkmale der Stichprobe	Thematischer Fokus
Haut (Hautzustand, Hauterkrankungen, chronische und schwerheilende Wunden)			
Hahnel et al. (2017)	n = 10 Pflegeheime	**IG 1**<br>Gesamt: n = 45 Bewohner:innen<br>Alter: 85 Jahre (SD: 8,3)<br>Weiblich: 56 %<br>**IG 2**<br>Gesamt: n = 44 Bewohner:innen<br>Alter: 83,7 Jahre (SD: 8,3)<br>Weiblich: 73 %<br>**KG**<br>Gesamt: n = 44 Bewohner:innen<br>Alter: 84 Jahre (SD: 8,9)<br>Weiblich: 68 %	Auswirkung von strukturierten Behandlungsplänen zur Hautpflege auf trockene Haut bei Bewohner:innen
Völzer et al. (2024)	n = 17 Pflegeheime	**IG**<br>Gesamt: n = 165 Bewohner:innen<br>Alter: 85,2 Jahre (SD: 7,3)<br>Weiblich: 68 %<br>**KG**<br>Gesamt: n = 149 Bewohner:innen<br>Alter: 85,6 Jahre (SD: 6,9)<br>Weiblich: 70 %	Auswirkung eines Hautpflegeprogramms zur Prävention und Behandlung häufiger Hauterkrankungen
Mobilität			

Referenz*	Anzahl der Einrichtungen	Merkmale der Stichprobe	Thematischer Fokus
Abraham et al. (2019)	n = 120 Pflegeheime	**IG 1**<br>Gesamt: n = 2.972 Bewohner:innen<br>Alter: 83,7 Jahre (SD: 9,7)<br>Weiblich: 71 %<br>**IG 2**<br>Gesamt: n = 2.523 Bewohner:innen<br>Alter: 83,5 Jahre (SD: 10,0)<br>Weiblich: 77 %<br>**KG**<br>Gesamt: n = 3.305 Bewohner:innen<br>Alter: 82,5 Jahre (SD: 10,5)<br>Weiblich: 73 %	Vermeidung von freiheitsentziehenden Maßnahmen
Cordes et al. (2021)	n = 4 Pflegeheime	**IG**<br>Gesamt: n = 26 Bewohner:innen<br>Alter: 82,7 Jahre (SD: 10,3)<br>Weiblich: 65 %<br>**KG**<br>Gesamt: n = 26 Bewohner:innen<br>Alter: 80,1 Jahre (SD: 11,4)<br>Weiblich: 62 %	Multikomponenten-Bewegungsintervention für Bewohner:innen, die nicht gehfähig sind, durchgeführt im Rollstuhl
Görres et al. (2016)	n = 33 Pflegeheime	*Nicht nach Studiengruppen differenziert berichtet*<br>Gesamt: n = 1.091 Bewohner:innen<br>Alter: 83,4 Jahre (SD: 9,4)<br>Weiblich: 74 %	Implementierung eines Expertenstandards zur Erhaltung und Förderung der Mobilität
Nguyen et al. (2023)	n = 35 Pflegeheime	**IG**<br>Gesamt: n = 301 Bewohner:innen<br>Alter: 85,6 Jahre (SD: 7,2)<br>Weiblich: 75 %<br>**KG**<br>Gesamt: n = 259 Bewohner:innen<br>Alter: 85,0 Jahre (SD: 7,2)<br>Weiblich: 71 %	Komplexe Intervention zur Verbesserung der Aktivitäten und Teilhabe von Bewohner:innen mit Gelenkkontrakturen

Referenz*	Anzahl der Einrichtungen	Merkmale der Stichprobe	Thematischer Fokus
Mundgesundheit			
Barbe et al. (2019)	n = 1 Pflegeheim	**IG**<br>Gesamt: n = 25 Bewohner:innen<br>Alter: 84 Jahre (SD: 10)<br>Weiblich: 76 %<br>**KG**<br>Gesamt: n = 25 Bewohner:innen<br>Alter: 83 Jahre (SD: 7)<br>Weiblich: 60 %	Wirksamkeit regelmäßigen Zähneputzens durch eine Zahnmedizinische Fachangestellte auf die Mundgesundheit der Bewohner:innen
Zenthöfer et al. (2016)	n = 14 Pflegeheime	*Nicht nach Studiengruppen differenziert berichtet*<br>Gesamt: n = 219 Bewohner:innen<br>Alter: 83,1 Jahre (SD: 9,0)<br>Weiblich: 69 %	Wirksamkeit einer Schulung von Pflegekräften sowie die Durchführung von Ultraschallbädern zur Reinigung von Zahnersatz auf die Mundgesundheit und Zahnersatzhygiene von älteren Menschen mit kognitiven und motorischen Einschränkungen
Schmerz			
Dräger et al. (2017)	n = 12 Pflegeheime	**IG**<br>Gesamt: n = 100 Bewohner.innen<br>Alter: 83,7 Jahre<br>Weiblich: 74 %<br>**KG**<br>Gesamt: n = 95 Bewohner:innen<br>Alter: 82,9 Jahre<br>Weiblich: 71 %	Intervention zur Optimierung des Schmerzmanagements und Bewertung der Auswirkungen auf die Schmerzintensität und schmerzbedingte Beeinträchtigungen der Funktionsfähigkeit
Schlaf			

Referenz*	Anzahl der Einrichtungen	Merkmale der Stichprobe	Thematischer Fokus
Dichter et al. (2024)	n = 24 Pflegeheime	**IG**<br>Gesamt: n = 126 Bewohner:innen<br>Alter: 84,8 Jahre (SD: 6,9)<br>Weiblich: 66 %<br>**KG**<br>Gesamt: n = 116 Bewohner:innen<br>Alter: 85,6 Jahre (SD: 7,1)<br>Weiblich: 74 %	Komplexe, nicht-pharmakologische Intervention zur Prävention und Reduktion von Schlafproblemen bei Menschen mit Demenz im Pflegeheim
Depressive Symptome			
Werner et al. (2017)	n = 2 Pflegeheime	**IG**<br>Gesamt: n = 62 Bewohner:innen<br>Alter: 83,1 (SD: 7,8)<br>Weiblich: 79 %<br>**KG**<br>Gesamt: n = 55 Bewohner:innen<br>Alter: 85,9 Jahre (SD: 7,8)<br>Weiblich: 56 %	Auswirkung von interaktiver Musiktherapie und gemeinschaftlichem Singen auf depressive Symptome von Bewohner:innen
Herausforderndes Verhalten bei Menschen mit Demenz			
Campbell et al. (2025)	n = 2 Pflegeheime	**IG 1**<br>Gesamt: n = 17 Bewohner:innen<br>Alter: 87,5 Jahre (SD: 7,9)<br>Weiblich: 82 %<br>**IG 2**<br>Gesamt: n = 13 Bewohner:innen<br>Alter: 87,6 Jahre (SD: 5,0)<br>Weiblich: 77 %<br>**KG**<br>Gesamt: n = 16 Bewohner:innen<br>Alter: 88 Jahre (SD: 4,4)<br>Weiblich: 69 %	Auswirkung von Musiktherapie auf herausfordernde Verhaltensweisen bei Menschen mit Demenz (BPSD - Behavioral and Psychological Symptoms of Dementia)

Referenz*	Anzahl der Einrichtungen	Merkmale der Stichprobe	Thematischer Fokus
Halek et al. (2020)	n = 12 Pflegeheime	**IG 1a**<br>Gesamt: n = 167 Bewohner:innen<br>Alter: 84 Jahre (SD: 8)<br>Weiblich: 67 %<br>**IG 1b**<br>Gesamt: n = 57 Bewohner:innen<br>Alter: 83 Jahre (SD: 9)<br>Weiblich: 65 %<br>**IG 2a**<br>Gesamt: n = 181 Bewohner:innen<br>Alter: 85 Jahre (SD: 8)<br>Weiblich: 79 %<br>**IG 2b**<br>Gesamt: n = 60 Bewohner:innen<br>Alter: 84 Jahre (SD: 6)<br>Weiblich: 73 %	Effekte von zwei demenz-spezifischen Fallkonferenzen auf die Prävalenz von herausfordernden Verhaltensweisen bei Menschen mit Demenz
Hillebrand et al. (2023)	n = 5 Pflegeheime	**IG**<br>Gesamt: n = 44 Bewohner:innen<br>Alter: 83,8 Jahre (SD: 7,3)<br>Weiblich: 82 %<br>**KG**<br>Gesamt: n = 46 Bewohner:innen<br>Alter: 84 Jahre (SD: 6,7)<br>Weiblich: 74 %	Effekte des individualisierten Musikhörens auf BPSD
Kratzer et al. (2022)	n = 26 Pflegeheime	**IG**<br>Gesamt: n = 60 Bewohner:innen<br>Alter: 85,0 Jahre (SD: 8,2)<br>Weiblich: 77 %<br>**KG**<br>Gesamt: n = 61 Bewohner:innen<br>Alter: 84,3 Jahre (SD: 5,1)<br>Weiblich: 75 %	Reduktion von BPSD und Verbesserung der Lebensqualität von Menschen mit einer schweren Demenz durch eine komplexe, nicht-pharmakologische Intervention

Referenz*	Anzahl der Einrichtungen	Merkmale der Stichprobe	Thematischer Fokus
Middelstädt et al. (2016)	n = 9 Pflegeheime	**IG**<br>Gesamt: n = 36 Bewohner:innen<br>Alter: 86,3 Jahre (SD: 4,7)<br>Weiblich: 83 %<br>**KG**<br>Gesamt: n = 35 Bewohner:innen<br>Alter: 86,5 Jahre (SD: 4,2)<br>Weiblich: 86 %	Effekt von kognitiver Stimulation auf herausforderndes Verhalten, Kognition, Lebensqualität und Aktivitäten des täglichen Lebens bei Menschen mit Demenz
O'Sullivan et al. (2022)	n = 10 Pflegeheime	**IG**<br>Gesamt: n = 80 Bewohner:innen<br>Alter: 85,4 Jahre (SD: 7,6)<br>Weiblich: 68 %<br>**KG**<br>Gesamt: n = 82 Bewohner:innen<br>Alter: 84,6 Jahre (SD: 6,6)<br>Weiblich: 79 %	Tablet-basierte Intervention zur Verringerung von Apathie bei Menschen mit Demenz
Beachtung individueller Präferenzen			
Götze et al. (2025)	n = 44 Pflegeheime	**IG**<br>Gesamt: n = 442 Bewohner:innen<br>Alter: 82,7 Jahre (SD: 10,2)<br>Weiblich: 68 %<br>**KG**<br>Gesamt: n = 450 Bewohner:innen<br>Alter: 83,6 Jahre (SD: 9,4)<br>Weiblich: 72 %	Auswirkung eines Advance Care Planning (ACP) - Programms auf die Bekanntheit und Einhaltung der Versorgungspräferenzen der Bewohner:innen
Medikation			

Referenz*	Anzahl der Einrichtungen	Merkmale der Stichprobe	Thematischer Fokus
Junius-Walker et al. (2021)	n = 44 Pflegeheime	**IG**<br>Gesamt: n = 402 Bewohner:innen<br>Alter: 84,7 Jahre (SD: 7,7)<br>Weiblich: 76 %<br>**KG**<br>Gesamt: n = 385 Bewohner:innen<br>Alter: 83,9 Jahre (SD: 8,1)<br>Weiblich: 71 %	Verbesserung der Arzneimittelsicherheit, u. a. durch Reduktion von potenziell inadäquaten Medikamenten (PIM) und/oder Neuroleptika
Lexow et al. (2022)	n = 3 Pflegeheime	**IG**<br>Gesamt: n = 107 Bewohner:innen<br>Alter: 86 Jahre (SD: k. A.)<br>Weiblich: 67 %<br>**KG**<br>Gesamt: n = 104 Bewohner:innen<br>Alter: 86 Jahre (SD: k. A.)<br>Weiblich: 72 %	Einmalige Überprüfung der Medikation durch Apotheker:innen auf Medikationsänderungen
Richter et al. (2019)	n = 37 Pflegeheime	**IG**<br>Gesamt: n = 439 Bewohner:innen<br>Alter: 84,0 Jahre (SD: 9,5)<br>Weiblich: 73 %<br>**KG**<br>Gesamt: n = 603 Bewohner:innen<br>Alter: 84,1 Jahre (SD: 9,1)<br>Weiblich: 74 %	Intervention zu Person-zentrierter Versorgung mit dem Ziel einer klinisch relevanten Verringerung der Verordnungen von Antipsychotika
Hospitalisierung			
Mazur et al. (2023)	n = 34 Pflegeheime	**IG**<br>Gesamt: n = 316 – 320 Bewohner:innen<br>Alter: 81,4 Jahre (12,2)<br>Weiblich: 69 %<br>**KG**<br>Gesamt: n = 321 – 323 Bewohner:innen<br>Alter: 82,1 Jahre (SD: 11,1)<br>Weiblich: 67 %	Einfluss interprofessioneller Zusammenarbeit auf die Hospitalisierungsrate der Bewohner:innen

Referenz*	Anzahl der Einrichtungen	Merkmale der Stichprobe	Thematischer Fokus
Tönnies et al. (2025)	n = 44 Pflegeheime	**IG**<br>Gesamt: n = 1.053 Bewohner:innen<br>Alter: 83 Jahre (SD: 10)<br>Weiblich: 70 %<br>**KG**<br>Gesamt: n = 680 Bewohner:innen<br>Alter: 80 Jahre (SD: 11)<br>Weiblich: 65 %	Effekt eines interprofessionellen Versorgungskonzepts (regionale Versorgungsteams bestehend aus Hausärzt:innen) auf die Hospitalisierungsrate von Bewohner:innen

*Referenzliste findet sich im Hauptmanuskript

Abkürzungen: ACP = Advance Care Planning, BPSD = Behavioral and Psychological Symptoms of Dementia, IG = Interventionsgruppe, k. A. = keine Angabe, KG = Kontrollgruppe, SD = Standardabweichung, PIM = potenziell inadäquate Medikamente
